# The design of a real-time formative evaluation of the implementation process of lifestyle interventions at two worksites using a 7-step strategy (BRAVO@Work)

**DOI:** 10.1186/1471-2458-12-619

**Published:** 2012-08-07

**Authors:** Debbie Wierenga, Luuk H Engbers, Pepijn van Empelen, Vincent H Hildebrandt, Willem van Mechelen

**Affiliations:** 1 Body@Work, Research Centre on Physical Activity, Work and Health, TNO-VUmc, Amsterdam, The Netherlands; 2 Department of Public and Occupational Health, EMGO + Institute for Health and Care Research, VU University Medical Centre, Amsterdam, The Netherlands; 3 Netherlands Organisation for Applied Scientific Research, TNO Expertise Centre Life Style, P.O. Box 2215, Leiden, 2301 CE, The Netherlands

**Keywords:** Implementation strategy, Process evaluation, Formative evaluation, Qualitative study, Worksite health promotion, Embedded scientist, Data triangulation

## Abstract

**Background:**

Worksite health promotion programs (WHPPs) offer an attractive opportunity to improve the lifestyle of employees. Nevertheless, broad scale and successful implementation of WHPPs in daily practice often fails. In the present study, called BRAVO@Work, a 7-step implementation strategy was used to develop, implement and embed a WHPP in two different worksites with a focus on multiple lifestyle interventions.

This article describes the design and framework for the formative evaluation of this 7-step strategy under real-time conditions by an embedded scientist with the purpose to gain insight into whether this this 7-step strategy is a useful and effective implementation strategy. Furthermore, we aim to gain insight into factors that either facilitate or hamper the implementation process, the quality of the implemented lifestyle interventions and the degree of adoption, implementation and continuation of these interventions.

**Methods and design:**

This study is a formative evaluation within two different worksites with an embedded scientist on site to continuously monitor the implementation process. Each worksite (i.e. a University of Applied Sciences and an Academic Hospital) will assign a participating faculty or a department, to implement a WHPP focusing on lifestyle interventions using the 7-step strategy. The primary focus will be to describe the natural course of development, implementation and maintenance of a WHPP by studying [a] the use and adherence to the 7-step strategy, [b] barriers and facilitators that influence the natural course of adoption, implementation and maintenance, and [c] the implementation process of the lifestyle interventions. All data will be collected using qualitative (i.e. real-time monitoring and semi-structured interviews) and quantitative methods (i.e. process evaluation questionnaires) applying data triangulation. Except for the real-time monitoring, the data collection will take place at baseline and after 6, 12 and 18 months.

**Discussion:**

This is one of the few studies to extensively and continuously monitor the natural course of the implementation process of a WHPP by a formative evaluation using a mix of quantitative and qualitative methods on different organizational levels (i.e. management, project group, employees) with an embedded scientist on site.

**Trial Registration:**

NTR2861

## Background

An unhealthy lifestyle (e.g. insufficient daily physical activity, unhealthy diet, smoking, high alcohol consumption and low levels of relaxation) is related to several chronic diseases with high prevalence rates in the Netherlands like cardiovascular diseases, diabetes mellitus type II, respiratory diseases (e.g. asthma and COPD), depression and certain types of cancer [1]. A well-known consequence of an unhealthy lifestyle is overweight [2,3]. Currently, 30.5% of the Dutch working population is overweight and an additional 6% is obese [4]. Furthermore, unhealthy and/or overweight employees show elevated sickness absence rates which significantly increase costs for the company [5]. To ensure long-lasting productivity of employees and to prevent work disability an important component is adopting and maintaining a healthy lifestyle [6,7].

The WHO states that the workplace directly influences the physical, mental, economic and social well-being of employees and in turn the health of their families, communities and society. Therefore, the workplace offers an ideal setting and infrastructure to support the promotion of health of a large audience [8]. Furthermore, growing evidence is found for the effectiveness of worksite health promotion programs (WHPP) that promote a healthy lifestyle in general [9-12]. Nevertheless, broad scale implementation of these effective WHPP in daily practice and across a wide range of settings often fails [13-17]. In order to improve the implementation of WHPPs into daily practice, it is important to shift the focus from effect evaluations to the evaluation of the implementation process. Hence key determinants of success and failure could be obtained and addressed in future implementation. For this purpose, traditional evaluation designs (i.e. randomized controlled trials) that focus on effect evaluations are not sufficient. These evaluation designs do not provide critical information on the implementation process. So other study designs are required, which focus more on observational strategies. The complementary use of a systematic and real-time formative evaluation within an controlled trial can create a dual style approach whereby critical information on the implementation process over time can be obtained [18,19]. A formative evaluation is an assessment that focuses on “the internal dynamics and actual operations of a program in order to understand its strengths and weaknesses and changes that occur in it over time” [18,20]. It gives researchers insight into program implementation over time and employs a mix of qualitative and quantitative techniques. Formative evaluations emphasize the need for real-time monitoring of the implementation process, but is very time consuming [18-20]. The amount of time that is needed to conduct a real-time formative evaluation could partially be the cause for the lack of such studies. However, investing time in effectiveness studies that are not used in daily practice and only include a posterior process evaluation that does not give insight into the important aspects of the implementation process, is also a waste of money.

Despite the lack of focus on studying the implementation process, researchers do acknowledge the fact that for improving the effectiveness and implementation of WHPP in practice, these programs should be systematically implemented in order to achieve successful implementation and continuation. For instance, Durlak and Dupre showed that the level of implementation (i.e. low or high implementation) affects the outcomes obtained by health promotion programs, whereby high implementation increased program success and could lead to greater effects on outcomes for participants [16]. In addition, implementation success is for an important part dependent on an adequate fit of the program with the specific organizational context (i.e. implementation context) in which the program is implemented [16]. The implementation context differs from one worksite to another because of inherent differences between worksites, which makes it difficult to implement effective WHPPs across different worksites [21]. In order to take the implementation context into account it is important to involve the target population (i.e. employees) and implementers within the worksites in the development phase of the WHPP and to keep them involved throughout the whole implementation process. This allows the worksite to incorporate and adjust the WHPP and implementation strategy to their specific needs, interests and the existing setting, thereby increasing the chances of implementation success [22].

**Figure 1:**
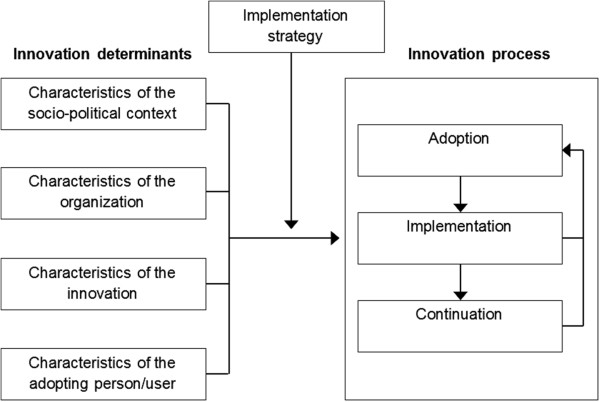
**Framework presenting the innovation process and related innovation determinants (Fleuren et al., **[25]**).**

Furthermore, in order to successfully implement WHPPs, programs need to pass through the four stages (i.e. dissemination, adoption, implementation and continuation) as stated in the diffusion of innovations theory [23]. Four main categories of innovation determinants may influence the transition process from one stage to the next as potential barriers or facilitators for implementation (see Figure 1): 1) characteristics of the socio-political context (e.g. fit with existing rules, regulations, and legislation), 2) characteristics of the organization (e.g. hierarchical structure, available expertise), 3) characteristics of the innovation (e.g. compatibility, relevance), and 4) characteristics of the adopting person/user (e.g. self-efficacy, degree of ownership) [24,25]. The above described theory provides the key elements that should be addressed when implementing a WHPP successfully [23,25]. However, this theory, along with other implementation theories, does not provide specific strategies or guidelines for implementation.

As such, a new and systematic 7-step implementation strategy was developed that incorporates most of the fore mentioned aspects for successful implementation. This strategy also aims to maintain the implemented programs over time [26,27]. The 7-step strategy is based on a ‘user-driven’ approach towards developing and implementing interventions that specifically address the capacities and needs of the target population at multiple organizational levels (i.e. management, project group, employees). User-driven within this context means that health objectives, interventions and implementation strategies are (co-)developed by members of the target population at different levels of the worksite. The 7-step strategy incorporates planning, implementation, evaluation and maintenance. The strategy ensures that the interventions will be tailored to the specifics of the worksite, thereby ensuring a fit with the implementation context. This increases possibilities for maintenance over time. The 7-step strategy has already been used in practice but whether this strategy is an effective and generic approach for developing and implementing WHPPs has never been studied systematically [28].

Therefore the present study, called BRAVO@Work, describes the formative evaluation of this 7-step strategy under real-time conditions by an embedded scientist, with the aim to evaluate and monitor whether this 7-step strategy is a useful and effective strategy to successfully develop and implement a WHPP at two worksites, with a focus on healthy lifestyle changes. Furthermore, we aim to gain insight into factors that either facilitate or hamper the implementation process, the quality of the implemented lifestyle interventions and the degree of adoption, implementation and continuation.

This article describes the design and framework for the formative evaluation of the natural course of the development, implementation and maintenance of BRAVO@Work.

## Methods and design

### Study design, population and setting

This study is a formative evaluation, alongside a controlled trial, within two different worksites with an embedded scientist on site to continuously monitor the implementation process. Each worksite (i.e. a University of Applied Sciences and an Academic Hospital) will assign a participating faculty (546 employees) or a department (635 employees) respectively, which will implement a WHPP using the 7-step strategy. Furthermore, both participating worksites will assign a control faculty/department that will not be allowed to participate in the implementation process and use of the 7-step strategy. Employees that are 18 years or older are eligible to participate in the study. Prior to data collection all employees will be informed about the study purposes, after which informed consent will be obtained. All data will be collected using qualitative (i.e. real-time monitoring and semi-structured interviews) and quantitative methods (i.e. process evaluation questionnaires), applying data triangulation. Except for the real-time monitoring, the data collection will take place at baseline and after 6, 12 and 18 months. Employees from different organizational levels of the worksite will be approached to actively participate in the project.

The study protocol has been approved by the Medical Ethics Committee of the University Medical Centre of Utrecht (Utrecht, the Netherlands).

### The 7-step strategy

**Figure 2:**
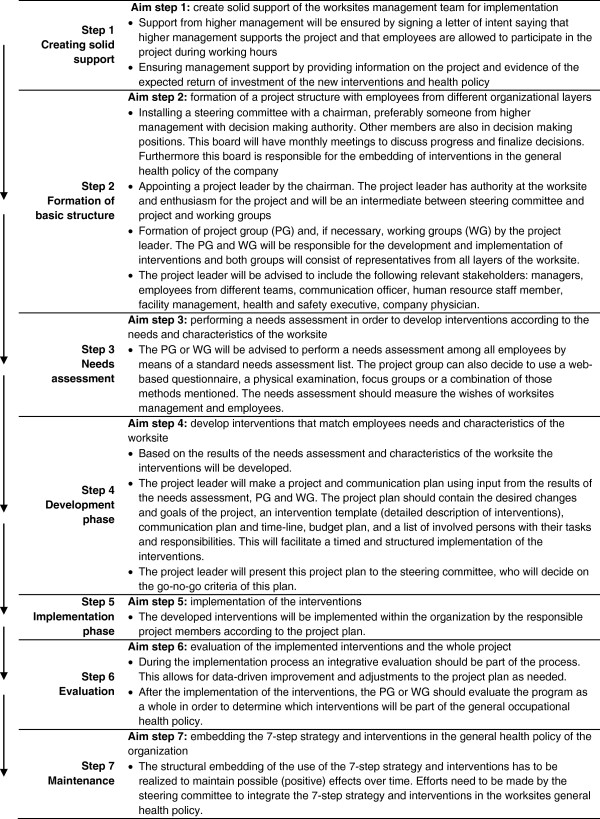
Outline of the 7-step strategy for implementation and continuation of a worksite health policy.

The 7-step strategy is based on a study in 1992 by Wynne and Clarkin and is supported by the European Foundation for the Improvement of living and Working Conditions [27]. The study of Wynne and Clarkin consisted of two phases. First they conducted a survey among almost 1500 European companies across seven countries questioning their health policies and other activities for worksite health promotion. Second, case studies of good practices were conducted to determine how these companies had organized activities for health promotion at the workplace and how they had integrated these activities in their general occupational health policy. The results of this study showed that the following five aspects were important when implementing a successful health policy at the workplace: A) **Needs assessment**: for the establishment of a health policy it is important that the wishes and needs of employees are analyzed. In this way the intervention activities can be developed according to their needs; B) **Participation**: key figures from different levels in the company’s organization need to be involved in the development and implementation of the health promotion program to create a solid support for the health policy. This can be done by means of working groups; C) **Flexibility**: health promotion programs are similar at some basic points. However, they are not standard programs, since a WHPP needs to fit the specifics of the workplace; D) **Integration**: the health promotion program needs to include activities that are both aimed at the individual employee and at the work environment; and E) **Multidisciplinary**: several experts in the fields of human resources, communication, health management, psychology and working environment need to be involved in the development and implementation to increase program effectiveness. These five aspects were translated by Wynne and Clarkin into a new and generic 7-step strategy for the systematic development and implementation of health promotion programs (interventions) at the workplace [26,27]. The implementation strategy consists of the following 7 steps: 1) creating solid support, 2) formation of basic structures, 3) performing a needs assessment, 4) development of the interventions and health policy, 5) implementation of the interventions, 6) evaluation of the implemented interventions, and 7) embedding the interventions in the general occupational health policy of the organization. A schematic description of the strategy is given in Figure 2.

The main aspect of this strategy is the active participation of relevant stakeholders (i.e. managers, employees, communications officer, human resources staff, facility management, health and safety executive, company physician) when passing through the 7 steps. Therefore, in step 2, it is recommended that representatives of employees and relevant stakeholders from the participating organizations should be asked to participate in the use and application of the 7-step strategy by taking part in a steering committee (SG) a project group (PG) or a working group (WG). The purpose of these various groups is explained in Figure 2, step 2. It is hypothesized that application of the 7-step strategy should ensure that developed and implemented interventions will be tailored to the needs of different stakeholders within the worksite. Furthermore it aims to facilitate the integration of successful interventions in company’s general occupational health policy.

In the BRAVO@Work study the 7-step strategy will be used to develop and implement interventions related to multiple lifestyle behaviors (i.e. BRAVO-interventions) at the worksite and to integrate these lifestyle interventions in the company’s general health policy. The way of use and the content of the 7-step strategy will be transferred to representatives within both participating worksites by the researchers. Since we aim to study the natural course of implementation, not the researchers but the representatives (SG, PG, WG) from both participating worksites themselves are set in the lead and made responsible for performing all actions needed according to the 7 steps. However, to ensure the quality of the interventions, the following guidelines are issued: A) only best evidence interventions that fit the worksite should be selected, B) the interventions should be related to at least two BRAVO lifestyle themes, and C) the interventions should involve an environmental component.

### Framework for evaluation of the implementation process

In order to systematically investigate and evaluate the implementation process the four main aspects of the theory described in the introduction (i.e. innovation determinants, adoption, implementation and continuation; Figure 1) are operationalized by using a combination of the framework of Steckler and Linnan for process evaluations and the RE-AIM framework [29-32]. After combining these framework, 8 descriptive components of the implementation process need to be operationalized and subsequently evaluated in this formative evaluation: 1) context, 2) recruitment, 3) reach, 4) dose delivered, 5) dose received, 6) fidelity, 7) satisfaction and 8) maintenance [29-32]. These components will be evaluated at three different levels within the participating worksites: 1) management level, 2) project group level and 3) employee level [24]. Table 1 presents the definition of all 8 components (including data collection method and evaluation level) of the formative evaluation which together will measure the degree of adoption, implementation and continuation of the 7-step strategy and the BRAVO-interventions within both participating worksites.

**Table 1:** Definition of the formative evaluation components at three organizational levels, including methods for data collection

**4 Main aspects of the implementation process**	**Evaluation component**	**Definition of evaluation component at relevant organizational levels**	**Data collection method**
**Adoption**	Recruitment	*Management level*	Monitoring
		Sources and procedures used to approach and attract worksites and management to become effective participants of the project	
		*Project group level*	Monitoring, questionnaire, interviews
		Sources and procedures used to approach and attract PG and WG members to become effective program members	
		*Employee level*	Monitoring, questionnaire, interviews
		Sources and procedures used to approach and attract employees for participation in BRAVO-interventions to become effective participants	
**Implementation**	Reach	*Project group level*	Monitoring, interviews
		Proportion of employees who were approached as PG or WG member	
		*Employee level*	Monitoring
		Proportion of employees who were approached for participation in BRAVO-interventions	
	Dose delivered	*Management level*	Monitoring
		Providing the 7-step strategy to worksites and PG and WG members	
		*Project group level*	Monitoring, questionnaire, interviews
		Proportion of intended BRAVO-interventions delivered or provided to employees by PG and WG	
	Dose Received	*Project group level*	Monitoring
		Proportion of companies and PG and WG members who received the 7-step implementation strategy	
		*Employee level*	Monitoring, questionnaire, interviews
		Proportion of employees who participated in each BRAVO-intervention	
	Fidelity	*Project group level*	Monitoring, questionnaire, interviews
		- Compliance to the 7 steps of the implementation strategy by Steering committee and PG -Compliance of PG to project implementation plan and quality of implementation of BRAVO-interventions	
	Satisfaction	*Management level*	Interviews
		Opinion/satisfaction about the project	
		*Project group level*	Questionnaire, interviews
		Opinion/satisfaction about the 7-step strategy	
		*Employee level*	Questionnaire, interviews
		Opinion/satisfaction about BRAVO-interventions	
**Continuation**	Maintenance	*Management level*	Monitoring, questionnaire, interviews
		Extend to which the developed BRAVO-interventions and 7-step strategy become routine and part of everyday culture and norms of the organization including the degree to which BRAVO-interventions are continued.	
**Implementation**	Context	*All levels*	Monitoring,
**Determinants**		Determinants of implementation which can either hinder or facilitate the implementation of the 7-step strategy and BRAVO-interventions. These determinants are subdivided within 4 main categories:	questionnaire, interviews
		1. Characteristics of the socio-political context	
		2. Characteristics of the organization	
		3. Characteristics of the innovation	
		4. Characteristics of the adopting person/user	

*PG = project group.

*WG = working group.

Adoption refers to the proportion of worksites and participants who will adopt the 7-step strategy and the BRAVO-interventions [25]. In order to successfully monitor adoption, we specifically examine recruitment. Implementation is the extent to which the intervention has been implemented and received by the intended audience. This will be assessed by examining reach, dose delivered, dose received, fidelity and satisfaction [28,29]. Continuation is the extent to which the program is sustained over time and has become part of everyday culture of the worksite. It will be operationalized within the component maintenance [25]. The four main categories of innovation determinants are operationalized within the component context and will be called implementation determinants. These implementation determinants could either facilitate or hamper implementation. Table 2 gives an overview of the implementation determinants that will be measured per main category.

**Table 2:** Implementation determinants measured in this study, sub dived per main category

**Main categories of implementation determinants**	**Measured implementation determinants**
*Characteristics of the socio-political context*	1. Willingness of participants to cooperate with the innovation
	2. Degree to which the participant is aware of the health benefits of the innovation
	3. The extent to which the innovation fits into existing rules, regulations and legislation
*Characteristics of the organization*	4. Decision making process and procedures in the organization: top-down or bottom-up
	5. Hierarchical structure: extent to which decision making process is formalized through hierarchical procedures
	6. Formal reinforcement by management to integrate the innovation into organizational policies
	7. Organizational size (number of employees): large, medium, small
	8. Functional structure (task oriented) versus product structure (output oriented)
	9. Staff turnover: high, average, low
	10. Degree of staff capacity in the organization or department that implements the innovation
	11. Available expertise, in relation to the innovation in the organization or department
	12. Number of potential users to be reached
	13. Financial resources made available for implementing the innovation
	14. Reimbursement for implementers/organizations to facilitate extra efforts in applying the innovation
	15. Other resources made available for implementing the innovation (e.g. equipment, manuals)
	16. Administrative support available to the implementers of the innovation
	17. Time available to implement the innovation
	18. Availability of staff responsible for coordinating implementation in the organization
	19. The implementers are involved in the development of the innovation
	20. Opinion leaders who influence opinions of others in the organization or department
	21. Cooperation with external partners with respect to the implementation of the innovation
*Characteristics of the adopting person/user*	22. Support from colleagues in implementing the innovation
	23. Support from other implementers within the project in implementing the innovation
	24. Support from their supervisors in the department with respect to the implementation of the innovation
	25. Support from higher management in the organization with respect to the implementation of the innovation
	26. Extent to which colleagues implement the innovation (modeling)
	27. Extent to which the implementer has the skills needed to implement the innovation
	28. Extent to which the implementer has the knowledge needed to implement the innovation
	29. Self-efficacy: confidence of the implementer to perform the behavior needed to implement the innovation
	30. Extent to which ownership by the implementer is perceived
	31. Extent to which the innovation first the perceived task orientation of the implementer
	32. Extent to which the implementer expects that the participant will cooperate with the innovation
	33. Extent to which the implementer expects that the participant will be satisfied with the innovation
	34. Extent to which the goals of the different implementers with respect to the innovation are contradictory
	35. Extent to which the implementer has ethical problems with the innovation
	36. Attitude of the implementer with respect to the innovation
	37. Outcome expectations of the implementer and participants with respect to the innovation
	38. Perceived social norm with respect to the innovation by colleagues and supervisors
	39. User directed performance feedback: formative or summative feedback
	40. Personal benefits for the implementers
	41. Extent to which the implementers work as a team
*Characteristics of the innovation*	42. Extent to which the procedures/guidelines of the innovation are clear
	43. Extent to which the procedures/guidelines are read by the implementers
	44. Extent to which the innovation is complete
	45. Extent to which the innovation is too complex to work with
	46. Information provided: sufficient, insufficient.
	47. Compatibility: degree to which the innovation is perceived as consistent with existing work procedures
	48. Triability: extent to which the innovation can be subjected to trial
	49. Relative advantage: extent to which the innovation is perceived as advantageous
	50. Observability: degree to which the results of the innovations are observable to the implementer
	51. Extent to which the innovation is appealing to use
	52. Relevance of the innovation for the participant: extent to which the innovation has added value
	53. Frequency of use of the innovation: high, low
	54. Image of the innovation in the organization: positive, negative

### Data collection procedure

All data will be derived from different sources and collected by means of different methods, both qualitative and quantitative (i.e. data triangulation). Data triangulation enables researchers to look for patterns in all collected data in order to develop an overall interpretation including multiple views on the implementation process [33,34]. The qualitative and quantitative data will complement each other and will thereby give insight into the natural course of implementation. The mix of qualitative and quantitative data will create a rich dataset for interpretation of the implementation process. The real-time formative evaluation was systematically planned prior to the start of the implementation within both participating worksites. This will enable us to collect all data on the 8 process components before, during and after the implementation of the lifestyle interventions in order to better understand the implementation process over time [18]. The evaluation is therefore an integral part of the ongoing implementation process.

The primary researcher will continuously monitor the use of the 7-step strategy and the implementation process of the BRAVO-interventions to gain insight into determinants of implementation and all 8 process components. The primary researcher will collect minutes from all project meetings and will document all communication (emails, letters, and phone calls) throughout the implementation process and use of the 7-step strategy within both participating worksites. These minutes and observations will be documented and structured by using monthly predetermined spread sheets. These spreadsheets will be constructed and structured according to our framework for evaluation and the three organizational levels (i.e. management, project group, and employees) of this study and will therefore contain information on all 8 process components related to the 7-step strategy, but also to the lifestyle interventions and the process of adoption, implementation and continuation.

Worksite management and team leaders will be asked in semi-structured telephone interviews at baseline (T0), after 6 months (T1) and after approximately 12 months (T2) for A) their experienced barriers and facilitators for the implementation of BRAVO-interventions (Context), B) whether they were aware of the project and if BRAVO-interventions were implemented (Fidelity and Dose delivered), and C) their expectations and satisfaction regarding the complete project (Satisfaction).

Data from project group (PG) and working group (WG) members will be collected by means of semi-structured interviews at T0, T1, T2 and after 18 months (T3). Furthermore, a process questionnaire will be distributed at T1 and T2. PG and WG members will be asked in the interviews and process questionnaire for A) their experienced barriers and facilitators for implementation related to the 7-step strategy and the implementation of the BRAVO-interventions (Context), B) for their expectations, experience and opinion about the use of the 7-step strategy and the implementation of the BRAVO-interventions (Satisfaction), C) for their adherence to the 7-step strategy and project plan (Fidelity), D) whether he or she implemented the intervention they were responsible for (dose delivered), E) which sources and procedures were used to approach and attract PG and WG members for participation in the project, and employees for participation in BRAVO-interventions (Recruitment), F) the intention to use the 7-step strategy and to continue the BRAVO-interventions in the future (Maintenance).

Employees from the participating faculty/department will be asked in semi-structured interviews at T0, T1 and T2 and subsequently by a process questionnaire at T1 and T2 for A) their experienced barriers and facilitators for the implementation of BRAVO-interventions (Context), B) whether they were aware of the project and the BRAVO-interventions (i.e. Reach) C) about their expectation and opinion of the project, BRAVO-interventions and ways of recruitment (Satisfaction and Recruitment), and D) their participation to BRAVO-interventions, including reasons for participation and non-participation (Dose received).

### Analysis

#### Analysis of qualitative data

All qualitative data will be analyzed with the software program for qualitative analyses ‘Atlas.ti’. All data that is systematically collected or observed during the study is considered to be data [35,36]. This means that not only the semi-structured, in-depth interviews, but also all data collected during the monitoring process by means of checklists and notes collected during attending project meetings will be regarded and analyzed as qualitative data. All collected data will be marked with a series of codes extracted from the text and from literature about the specific topic (i.e. open coding: describes the content of the material). The codes will be grouped into concepts in order to make them more workable (i.e. selective coding: refer to central concepts underlying the descriptive codes) [35,36]. These concepts will be categorized to form the basis for the creation of a theory behind the data by using tables and matrices to identify and compare concepts (i.e. theoretical coding: identify patterns and relationships between concepts). All qualitative data will be presented with representative quotes, which cannot be traced back to individual persons.

#### Statistical analysis of quantitative data

For our main objective we aim to observe trends in self-reported and observed implementation of the use of the 7-step strategy and of the implemented lifestyle interventions. That is, at level of project initiation we will examine the results of the project group. At the employee level, we examine self-reported exposure, acceptance and use of lifestyle program components. Mixed-effects logistic regression will be used to examine trends. A two-tailed significance level of p < 0.05 is considered to be statistically significant. This analysis will allow for the use of probabilistic or dichotomous data, and will take into account that repeated observations are nested within individuals. Within this context we will also examine the determinants that may explain exposure, acceptance and uptake level. Furthermore we will analyze demographic variables of non-responders compared to responders the questionnaire. Non-responders are employees who received the questionnaire but did not return it. Analyses will be performed with SPSS 20.0 (SPSS Inc. Chicago, Illinois, USA).

## Discussion

The purpose of this article was to describe the design and framework for the formative evaluation of the natural course of the development and implementation of BRAVO@Work. Additionally, the seven steps of the applied implementation strategy were presented. This 7-step strategy was designed to successfully develop and implement a WHPP at two work sites, with a focus on healthy lifestyle changes.

The rising call to improve the translation of research into daily practice has created a need for a shift in focus towards the evaluation of the implementation process of interventions, rather than the current focus on effect evaluations. Consequently, one of the main strengths of the BRAVO@Work study is that, to our knowledge, this is one of the few studies that systematically monitors and evaluates the natural course of the implementation process prior to, during and after implementation by means of a real-time formative evaluation within a controlled trial. The formative evaluation of the implementation process will be conducted on multiple organizational levels and is systematically planned prior to the start of the implementation within both participating worksites. A well-planned and structured evaluation of the implementation process can provide critical information that could help explain study outcomes on the effectiveness of an intervention but most important, it could provide a base for enhancing program maintenance [37-40]. The data gathered are essential for generalizing the strategy to other settings and thereby improving the translation of research into daily practice by determining the factors that either facilitate or hamper implementation. The real-time monitoring allows us to gain insight into possible changes over time in the determinants of implementation, such as the attitude towards the 7-step strategy/interventions and the intentions to continue. It will allow us to better understand possible failure points in the 7-step strategy. These results will be used to enrich the 7-step strategy, resulting in an implementation strategy suitable for practice. In addition, the 7-step strategy will be evaluated in two different worksites (i.e. education and healthcare), with different organizational structures, cultures, work forces and tasks. This enables us to gain insight into the generalizability of the 7-step strategy across different worksites.

As mentioned before the best method to open the ‘black box’ of the implementation process of interventions is to place the researcher on site as an embedded observer (‘fly on the wall’). Our approach fits well with recent calls, such as that of Wandersman et al., (2008) which highlight the need for user-based rather than source-based approaches. Source-based programs follow a linear sequence, meaning that the innovation is directly transferred and implemented from the perspective of the developers (I.e. source) to the users without adaptation to the specific setting in which the innovation will be used [22]. Alternatively, Wandersman et al., (2008) have focused on a user-based model, whereby interventions and implementation strategies are developed by the source but implemented based on the awareness of needs and opportunities for change from the user. This calls for alternative research designs, such as the design of the BRAVO@Work study. Our approach ensures that the dynamic process of implementation is captured by means of data triangulation, in which multiple methodologies are used to examine WHPP assessment, development, implementation and continuation.

Another strength of this study is that the formative evaluation will allow us to gain insight into the fit of 7-step strategy and the implemented lifestyle interventions with the worksite. This is an important aspect of the evaluation since the 7-step strategy and literature emphasizes the need for a fit with the worksite in order to achieve successful implementation.

A final and most important strength of this study is the use of a mixed methods approach (i.e. observation, monitoring, questionnaires and semi-structured interviews), accompanied by collecting data at different organizational levels in the formative evaluation of the implementation process. This data triangulation is a way of ensuring the integrity of the data since multiple views on the implementation process are mapped [34].

However, limitations of this study can also be mentioned. First selection bias due to a selective response could occur. Healthy employees are more likely to fill out the questionnaires and to participate in interviews and interventions. This is the case with instruments that address health behaviors [41]. However, one might hypothesize that this will be less of an issue in formative evaluations, because stating your (positive or negative) opinion on the interventions does not deal with (changing) your poor or good behavior. Hence, it is less personal. To address this potential problem we will analyze demographic variables of non-responders and invite them for interviews. A second limitation might be that members of the project group (i.e. employees) might be a poor representation of all employees working at the worksite. Ideally, employees participating in one of the three groups are ambassadors of the project at their faculty/department. However, due to the nature of this study we do not control the selection of employees for participation in these groups. Instead, we could only advice the project leader to include employees from all relevant faculties/departments as stated in the 7-step strategy.

Since we will conduct a formative evaluation alongside a controlled trial both participating worksites will assign a control faculty/department that will not be allowed to participate in the implementation process. This will allow us to collect data on the effectiveness of the implemented lifestyle interventions by means of a web-based questionnaire distributed in the intervention faculty/department (i.e. department working with the 7-step strategy) and in the control faculty/department. In doing so we will be able to link the outcomes on the effectiveness to the implementation process and will give us insight into the quality of the implemented lifestyle interventions and possibly the separate effects of each implemented lifestyle intervention [30,42-45].

When BRAVO@Work proves to be successful, the 7-step strategy will be adjusted if necessary and then disseminated nationwide by the Dutch Institute for Sport and Physical Activity, providing companies with an effective strategy to develop and implement a lifestyle policy as part of their health management.

## Abbreviations

WHPP: Worksite Health Promotion Program; SG: Steering committee; PG: Project Group; WG: Working Group.

## Competing interests

The author(s) declare that they have no competing interests.

## Authors’ contributions

LE and VH wrote the initial study protocol and were involved in preparations for the study. DW was responsible for drafting the paper. All authors commented on the draft versions. All authors have read and approved the final version of the manuscript.

## Pre-publication history

The pre-publication history for this paper can be accessed here:

http://www.biomedcentral.com/1471-2458/12/619/prepub
